# Development and evaluation of multimodal AI for diagnosis and triage of ophthalmic diseases using ChatGPT and anterior segment images: protocol for a two-stage cross-sectional study

**DOI:** 10.3389/frai.2023.1323924

**Published:** 2023-12-08

**Authors:** Zhiyu Peng, Ruiqi Ma, Yihan Zhang, Mingxu Yan, Jie Lu, Qian Cheng, Jingjing Liao, Yunqiu Zhang, Jinghan Wang, Yue Zhao, Jiang Zhu, Bing Qin, Qin Jiang, Fei Shi, Jiang Qian, Xinjian Chen, Chen Zhao

**Affiliations:** ^1^Department of Ophthalmology, Fudan Eye & ENT Hospital, Shanghai, China; ^2^Department of Ophthalmology, The First Affiliated Hospital, Zhejiang University School of Medicine, Hangzhou, Zhejiang, China; ^3^Laboratory of Myopia, Chinese Academy of Medical Sciences, Shanghai, China; ^4^NHC Key Laboratory of Myopia, Fudan University, Shanghai, China; ^5^School of Basic Medical Sciences, Fudan University, Shanghai, China; ^6^School of Public Health, Fudan University, Shanghai, China; ^7^Medical Image Processing, Analysis, and Visualization (MIVAP) Lab, School of Electronics and Information Engineering, Soochow University, Suzhou, China; ^8^The Affiliated Eye Hospital, Nanjing Medical University, Nanjing, China; ^9^Department of Ophthalmology, Suqian First Hospital, Suqian, China; ^10^The Fourth School of Clinical Medicine, Nanjing Medical University, Nanjing, China; ^11^State Key Laboratory of Radiation Medicine and Protection, Soochow University, Suzhou, China

**Keywords:** multimodal AI, ophthalmic diseases, ChatGPT, anterior segment images, cross-sectional study, protocol, prompt engineering

## Abstract

**Introduction:**

Artificial intelligence (AI) technology has made rapid progress for disease diagnosis and triage. In the field of ophthalmic diseases, image-based diagnosis has achieved high accuracy but still encounters limitations due to the lack of medical history. The emergence of ChatGPT enables human-computer interaction, allowing for the development of a multimodal AI system that integrates interactive text and image information.

**Objective:**

To develop a multimodal AI system using ChatGPT and anterior segment images for diagnosing and triaging ophthalmic diseases. To assess the AI system's performance through a two-stage cross-sectional study, starting with silent evaluation and followed by early clinical evaluation in outpatient clinics.

**Methods and analysis:**

Our study will be conducted across three distinct centers in Shanghai, Nanjing, and Suqian. The development of the smartphone-based multimodal AI system will take place in Shanghai with the goal of achieving ≥90% sensitivity and ≥95% specificity for diagnosing and triaging ophthalmic diseases. The first stage of the cross-sectional study will explore the system's performance in Shanghai's outpatient clinics. Medical histories will be collected without patient interaction, and anterior segment images will be captured using slit lamp equipment. This stage aims for ≥85% sensitivity and ≥95% specificity with a sample size of 100 patients. The second stage will take place at three locations, with Shanghai serving as the internal validation dataset, and Nanjing and Suqian as the external validation dataset. Medical history will be collected through patient interviews, and anterior segment images will be captured via smartphone devices. An expert panel will establish reference standards and assess AI accuracy for diagnosis and triage throughout all stages. A one-vs.-rest strategy will be used for data analysis, and a *post-hoc* power calculation will be performed to evaluate the impact of disease types on AI performance.

**Discussion:**

Our study may provide a user-friendly smartphone-based multimodal AI system for diagnosis and triage of ophthalmic diseases. This innovative system may support early detection of ocular abnormalities, facilitate establishment of a tiered healthcare system, and reduce the burdens on tertiary facilities.

**Trial registration:**

The study was registered in ClinicalTrials.gov on June 25th, 2023 (NCT 05930444).

## Introduction

Artificial intelligence (AI) technology has been generally applied to medical research and practice. Deep learning models, trained on large datasets, have demonstrated impressive capabilities for diagnosis, especially in the fields of image analysis within radiology (van Leeuwen et al., 2021), pathology (Niazi et al., 2019), and dermatology (Phillips et al., 2019). In ophthalmology, AI studies using image data such as fundus images, anterior segment images, optical coherence tomography and computed tomography images have achieved high accuracy in diagnosing glaucoma (Buisson et al., 2021; Akter et al., 2022), age-related macular degeneration (Yan et al., 2021; Chen et al., 2022), diabetic retinopathy (Son et al., 2020; Li et al., 2022), thyroid-associated ophthalmopathy (Shao et al., 2023a), corneal diseases (Gu et al., 2020; Fang et al., 2022; Tiwari et al., 2022), and ocular tumors (Huang et al., 2022; Shao et al., 2023b). Despite these achievements, an obvious limitation of image-based diagnosis is its inability to consider a patient's medical history, which restricts a comprehensive understanding of the patient's condition. To address this limitation, there is a growing need for a multimodal AI system that integrates text and image information to enhance diagnostic performance. Recently, large language models (LLM) have shown great potential in comprehending and reasoning about textual information (Xie et al., 2023). These tools can be utilized in diagnostic AI models to enable textual interaction with patients and obtain in-depth information about the disease to aid in diagnosis.

ChatGPT, a web-application created by OpenAI, utilizes Generative Pre-trained Transformer (GPT) models GPT-3.5 and GPT-4.0, and has brought a new wave to AI research in medicine (Thirunavukarasu et al., 2023). GPT possesses a wide range of knowledge and language capabilities acquired through extensive pre-training on a large scale of text database (Thirunavukarasu et al., 2023). These remarkable human-computer interaction capabilities, exemplified by ChatGPT, allow it to generate natural-language text responses based on user input, indicating the potential application of ChatGPT in online consultations and diagnosis (Ayers et al., 2023). Recently, Gilson et al. (2023) and Kung et al. (2023) reported that ChatGPT possessed sufficient medical knowledge reservoir and reasonable logic loop to pass the United States Medical Licensing Exam (USMLE), highlighting its potential use in medical practice. Berg et al. (2023) generated a ChatGPT-based research and successfully triaged patients at the emergency department. Several studies have also made attempts to utilize ChatGPT for ophthalmic disease diagnosis. For instance, ChatGPT showed good performance in answering ophthalmology board-style questions, indicating its possession of specialized ophthalmic knowledge (Cai et al., 2023). Lyons et al. (2023) compared the triage performance of different AI chatbots in classifying ophthalmic diseases into four urgency categories (self-care, non-urgent, semi-urgent, or urgent), and ChatGPT is reported to be the most prominent one. Rojas-Carabali et al. and Delsoz et al. evaluated the diagnostic accuracy of ChatGPT in uveitis and glaucoma, respectively. Both studies demonstrated promising results for these specific diseases. However, the overall diagnostic accuracy of ChatGPT for various ophthalmic diseases still remains unsatisfactory (Delsoz et al., 2023; Rojas-Carabali et al., 2023).

Although ChatGPT has shown improved diagnostic performance compared to previous natural language models, it still has limitations. This web application is unable to autonomously collect medical history, which therefore restricts its application scenarios and diagnostic accuracy (Howard et al., 2023). In our study, we will implement an innovative dynamic prompt engineering technology and consultation process framework to enable intelligent consultations. This will address the limitation of LLM, which lacks the capability to inquire about patients' primary complaints and symptoms. Moreover, the performance of ChatGPT is unsatisfactory when it comes to questions involving images (Suhag et al., 2023). Recognizing the challenges of low diagnostic accuracy due to the absence of image information in language model consultations, it is necessary to integrate deep learning algorithms for image analysis. This integration allowed our system to effectively handle both text-based consultations and photo image data, leading to significant improvements in the diagnosis of certain ophthalmic diseases.

In our study, we aim to develop a multimodal AI system for the diagnosis and triage of ophthalmic diseases by integrating image analysis with ChatGPT and prompt engineering technology. We will conduct a two-stage cross-sectional study to assess the performance of this multimodal AI system. This innovative multimodal AI system holds the potential for practical real-world applications, such as aiding local communities in the early detection of ocular abnormalities, facilitating the establishment of a tiered healthcare system, and reducing the burden on tertiary healthcare facilities.

## Methods and analysis

### Objectives

**Figure 1:**
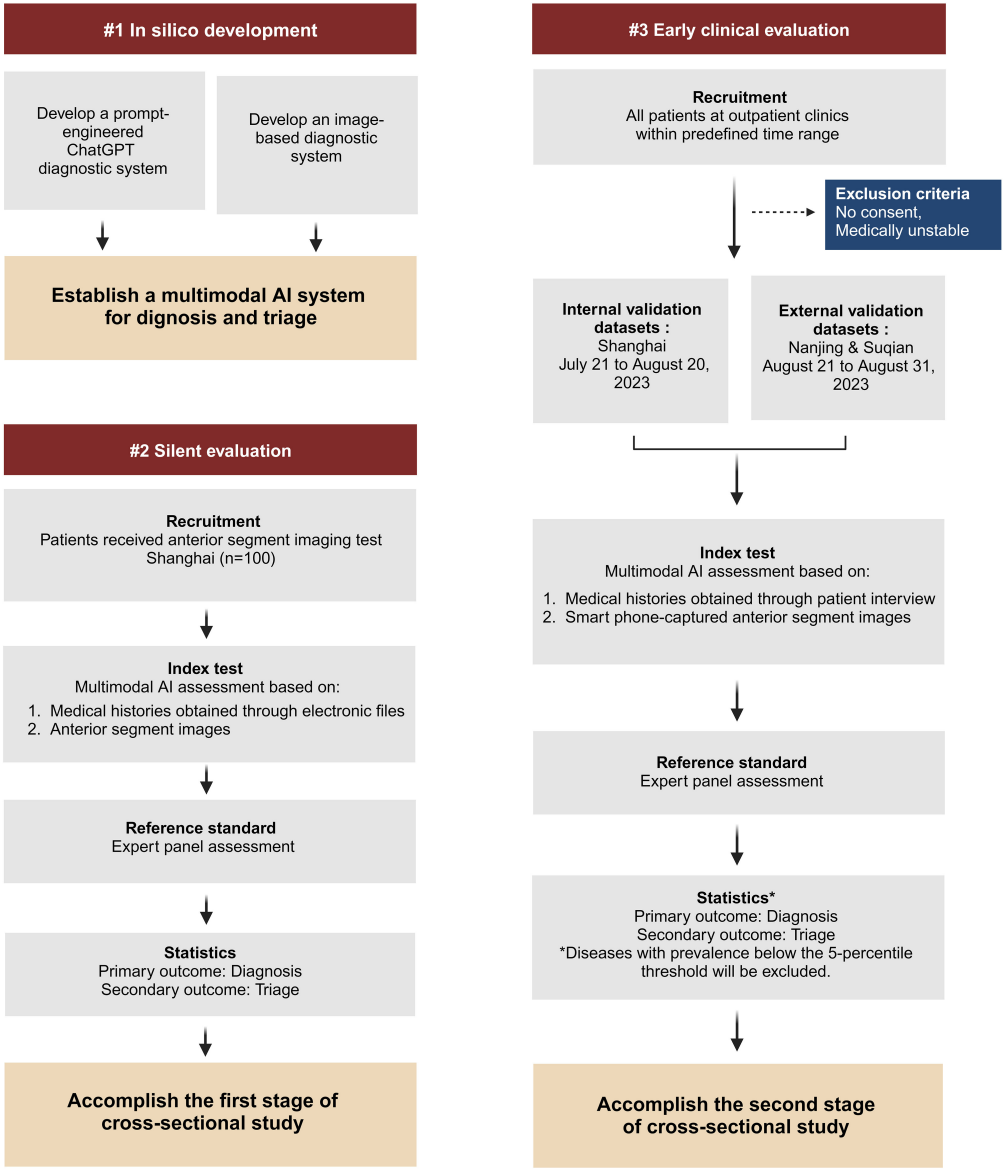
Flowchart of the multimodal AI study.

Our study consists of a development stage and two evaluation stages of cross-sectional study (Figure 1):

(1) During the *in silico* development stage, we aim to develop a multimodal AI system using ChatGPT and anterior segment images captured in medical facility to facilitate the diagnosis and triage of ophthalmic diseases.
(2) During the first evaluation stage (silent evaluation), we aim to evaluate the AI system's performance based on medical histories obtained through observation without patient interaction and anterior segment images captured in medical facility.
(3) During the second evaluation stage (early clinical evaluation), we aim to evaluate the AI system's performance in outpatient clinics based on medical histories obtained through patient interview and smartphone-captured anterior segment images.

### Hypotheses

For *in silico* development, our primary goal is to achieve a sensitivity of ≥90% and a specificity of ≥95% for diagnosing ophthalmic diseases. As a secondary outcome, we expect a positive predictive value of ≥90% and a negative predictive value of ≥95% for subspecialty triage of ophthalmic diseases.

For silent evaluation, the primary outcome is to achieve a sensitivity ≥ 85% and specificity ≥ 95% for diagnosing ophthalmic diseases. The secondary outcome is to achieve a positive predictive value ≥ 85% and a negative predictive value ≥ 95% for subspecialty triage of ophthalmic diseases.

For early clinical evaluation, we anticipate the primary outcome to reach a sensitivity ≥ 75% and specificity ≥ 95% for diagnosing ophthalmic diseases. For the secondary outcome, we expect a positive predictive value of ≥80% and a negative predictive value of ≥95% for subspecialty triage of ophthalmic diseases.

### Study design

#### In silico development

**Figure 2:**
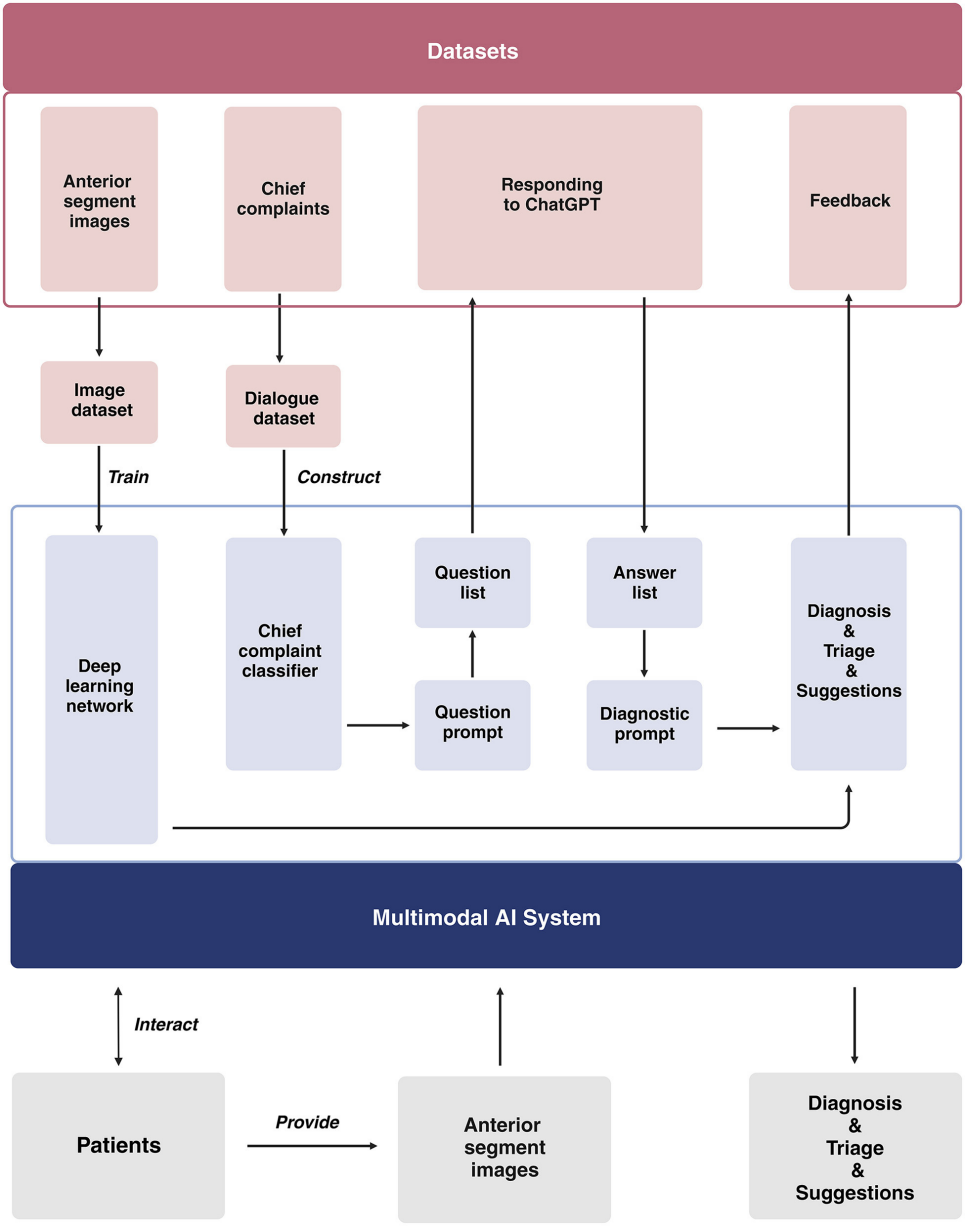
Flow diagram of the technical details of the multimodal AI system.

**Figure 3:**
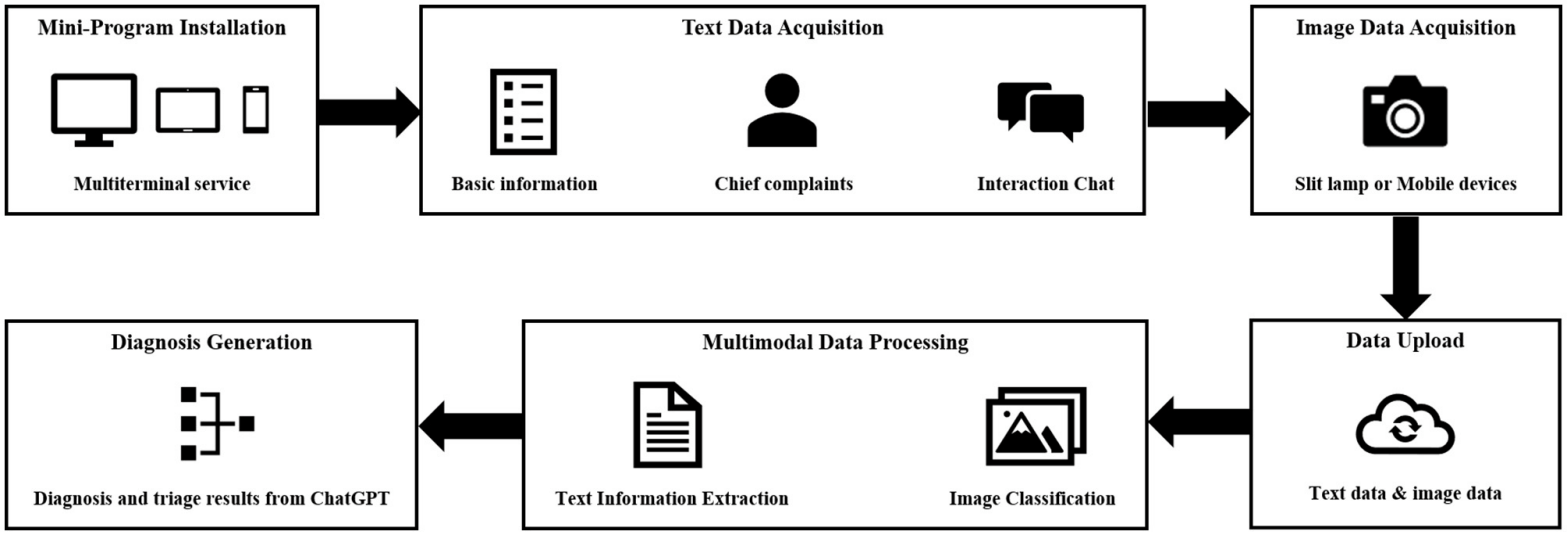
Overview of the multimodal AI framework and functioning.

Our study will be conducted across three distinct centers located in Shanghai, Nanjing, and Suqian. The development of the multimodal AI system takes place in Shanghai. We will gather medical dialogues (at least 20 dialogues for each subspecialty) from 10 ophthalmic subspecialties, which include general ophthalmology, refractive diseases, strabismus, cornea, cataract, glaucoma, retina, neuro-ophthalmology, oculoplastics and orbital diseases, and ophthalmic emergencies (Supplementary Tables 1, 2). We will utilize these dialogues for prompt engineering to guide the ChatGPT diagnostic system. The brief process is as follows (Figure 2): When users input their chief complaints, the system will categorize them using a chief complaint classifier. Once classified, the system will automatically generate a tailored question list for the user and output each question from the list. After receiving the user's responses, both the chief complaints and all answers will be consolidated into the diagnostic prompt, which ultimately produces the user's diagnosis and triage results. Once the interactive system is developed, virtual cases (at least 50 cases for each disease) representing the top 1–5 diseases in each subspecialty will be introduced to assess diagnostic efficacy. For the diseases that fail to meet the predefined performance thresholds (sensitivity ≥ 90%, specificity ≥ 95%), we will develop an image-based diagnostic system tailored to the cases that have anterior segment images. Subsequently, we will integrate these components to establish a multimodal AI system, with a primary focus on image-based diagnosis, for the comprehensive evaluation of ophthalmic diseases (Figure 3).

#### Silent evaluation

The multimodal AI system will be integrated into a mobile mini-program to enable the diagnosis and triage of ophthalmic diseases. This mini-program will undergo a silent evaluation phase, conducted as a cross-sectional study, in Shanghai (STROBE checklist for cross-sectional study is detailed in Supplementary material 1). A team of researchers will collect medical history on the mobile mini-program in outpatient clinics without intervening in the medical inquiry process. For patients who have undergone anterior segment imaging examinations (Supplementary Table 3), the researchers will provide the corresponding anterior segment images and respond to queries generated by the AI system, utilizing information extracted from the medical dialogues. In cases where no relevant information is available for a specific query, the researcher will denote it as “no information available”.

#### Early clinical evaluation

**Figure 4:**
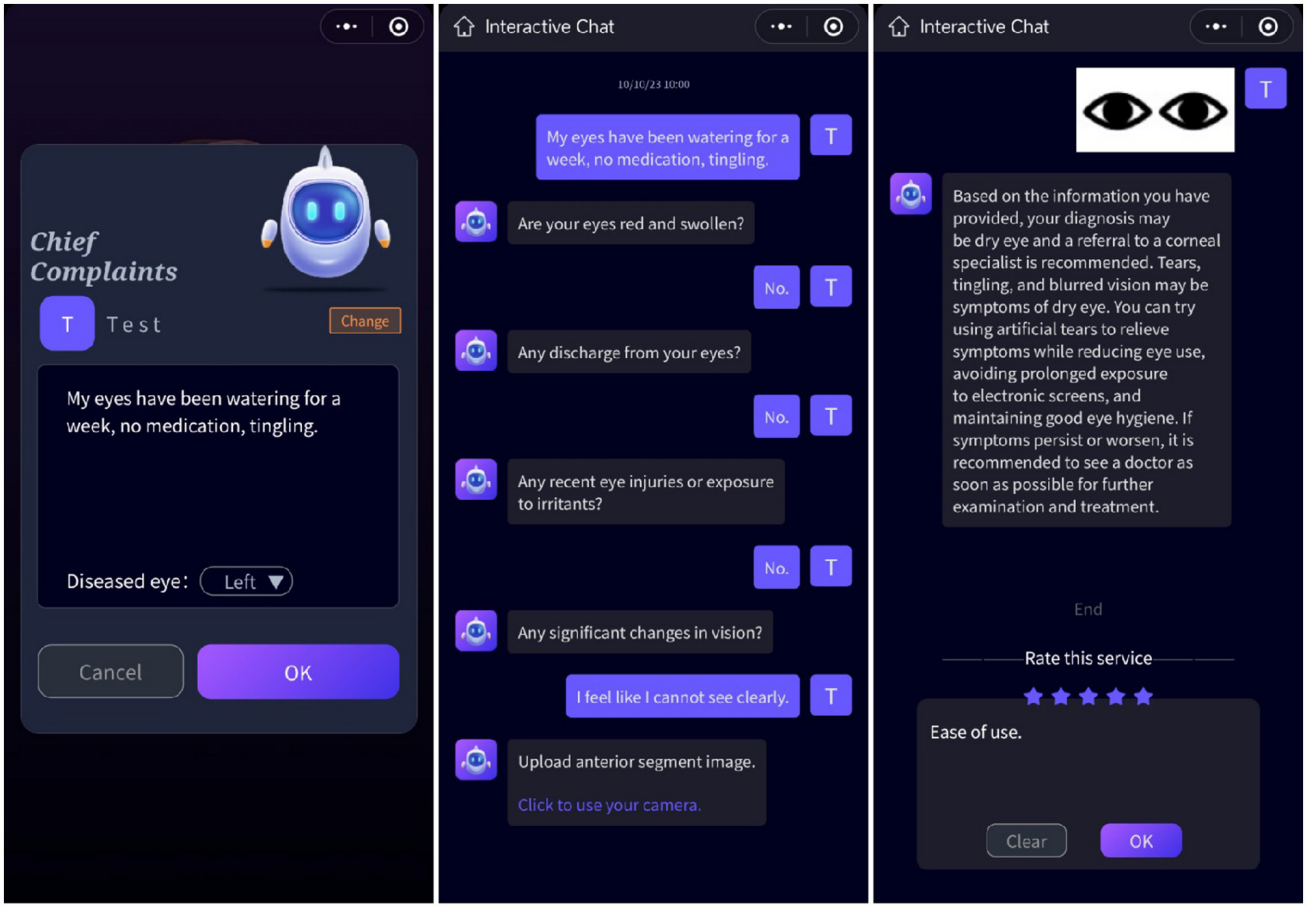
User interface. Users input their chief complaints in the initial interface. Following this, dialogues between ChatGPT and users are generated once the page transitions. Anterior segment images are obtained after the conversation concludes.

The mobile mini-program will be upgraded to analyze anterior segment images obtained by smartphone-based devices. An early clinical evaluation of this upgraded program will be simultaneously conducted as a cross-sectional study at three sites: the Eye & ENT Hospital of Fudan University (Shanghai), the Affiliated Eye Hospital of Nanjing Medical University (Nanjing), and Suqian First People's Hospital (Suqian). During the study, researchers will interact with patients to collect necessary information for completing the queries generated by the AI system. Anterior segment images will be captured for each patient by researchers using smartphones (Figure 4). The images should be captured by the macro lens of the smartphone. The positions of both eyes should align with the eye locations specified on the smartphone screen. The images should be large enough to provide a clear view of the affected area of the diseased eye. For all stages, the reference standards of disease diagnoses and subspecialty triage will be determined by an expert panel comprising ophthalmic specialists with a minimum of 5 years of clinical experience. Upon the completion of these data collections, a separate expert panel will assess the accuracy of AI intelligent diagnosis and triage.

### Eligibility criteria

Medical dialogues in the development stage should comply with the following criteria: (1) Inclusion criteria: We will include dialogues from both initial consultations and follow-up visits that provide adequate medical history information while minimizing the inclusion of physical examination details. The diagnosis should correspond to diseases ranked within the top 5 outpatient cases for each subspecialty, based on data from the Eye & ENT Hospital of Fudan University over the past 3 years. (2) Exclusion criteria: we will exclude diseases that are diagnosed or triaged solely based on physical examinations.

Anterior segment images in the development stage should comply with the following criteria: (1) Inclusion criteria: Individuals should have a documented medical history and diagnosis; Following a review of the medical history, both the diagnosis and subspecialty triage should have been confirmed according to the reference standard, as specified below. (2) Exclusion criteria for anterior segment images: photos with insufficient lighting or facial covering.

In the silent evaluation and early clinical evaluation stages, all cases should adhere to the following criteria: (1) Inclusion criteria: informed consent must be obtained from all participants; participants should have specific concerns or issues directly related to their eyes; for patients who are unable to provide informed consent due to incapacity or are underage or otherwise vulnerable, consent from must be obtained from their legal guardians and legal guardians are required to assist in the collection of medical history and images; each participant's diagnosis and subspecialty triage must be validated using a reference standard as detailed below. (2) Exclusion criteria: cases where the diagnosis and triage results fail to generate due to a program error; patients who, in the opinion of the attending physician or clinical study staff, are too medically unstable to participate in the study safely; diseases with prevalence below the 5-percentile threshold are not included.

### Recruitment

For the *in silico* development stage, all cases will be recruited at the Eye & ENT Hospital of Fudan University. Specifically, researchers will record doctor-patient conversations at each subspecialty clinic. The conversations will undergo a curation process that involves the removal of filler words and irrelevant content, resulting in the creation of medical dialogues. Eligible medical dialogues will be continuously enrolled from June 1 to July 1, 2023. Regarding the anterior segment images, researchers will browse medical records and consistently gather images related to the top 1–5 diseases in each subspecialty from January 1, 2020 to July 1, 2023. The initial sample size consists of 20 dialogues per specialty and 50 images per disease, which is then adjusted according to the AI system's performance.

For the silent evaluation stage, data will be continuously gathered in Shanghai from July 10 to July 15, 2023, with a sample size of 100 cases. Two pre-trained researchers will serve as observers in outpatient clinics, ensuring they do not interfere with standard management procedures. Upon obtaining the patients' consent (as detailed in Supplementary material 2), the researchers will collect the patients' medical information and enter it into the system. For patients who are prescribed with anterior segment imaging test by outpatient clinic doctors, the researchers will collect the images from the electronic medical system.

For the early clinical evaluation stage, eligible participants will be continuously recruited at three distinct sites. The Shanghai site serves as an internal validation dataset, with data collection spanning from July 21 to August 20, 2023. The Nanjing and Suqian sites function as external validation dataset, with data collection scheduled from August 21 to August 31, 2023. Each center will be staffed with two pre-trained researchers. These researchers will be responsible for patient recruitment across various subspecialty clinics within their respective centers. Once patients provide their consent (as detailed in Supplementary material 4) to participate in the study, the researchers will gather medical history and acquire anterior segment images. Following this, outpatient physicians will proceed with clinical diagnosis and treatment.

### Reference standard

The reference standard is determined by an expert panel comprising five senior consultants (with a minimum of 5 years of clinical experience). A consensus of diagnosis and triage is required from at least three out of these five experts after a comprehensive review of the patients' medical histories. In cases where expert consensus cannot be achieved, patients will be asked to attend follow-up appointments, during which additional examinations may be recommended. If, after 3 months of follow-up, a definitive diagnosis remains elusive, the patient will be excluded from the study.

### Index test

During the *in silico* development stage, the index test refers to either the ChatGPT-based medical inquiry system or the image-based system utilizing anterior segment images acquired via slit-lamp or camera in medical facility for ophthalmic disease diagnosis and triage. During the silent evaluation stage, the index test refers to a multimodal AI system that integrates the aforementioned components for diagnosis and triage. During the early clinical evaluation stage, the index test refers to an upgraded multimodal AI system capable of analyzing anterior segment images obtained through smartphones.

### Outcome measure

The primary outcome is the correctness of the AI system's diagnosis, and the secondary outcome is the correctness of the AI system's triage decisions. The above assessment takes place by comparing the AI-generated results with the reference standards established by expert panels:

(1) Judged as correct: when the system's results are entirely consistent with the reference standard; when the system's results fall within the range of the reference standard.
(2) Judged as incorrect: when there is no overlap between the system's results and the reference standard; when the system fails to provide any diagnostic or triage information.
(3) In the following scenarios, additional discussion and assessment are required. A consensus is considered to be reached when at least 2 out of 3 experts are in agreement: when the reference standard includes the system's results; when there is partial overlap between the system's results and the reference standard.

In addition to assessing accuracy, we will also evaluate the quality, empathy, and patient satisfaction of the AI system's responses. Three independent evaluators (licensed healthcare professionals), who are blinded to the accuracy of the responses, will judge both “the quality of information provided” (very poor, poor, acceptable, good, or very good) and “the empathy of information provided” (not empathetic, slightly empathetic, moderately empathetic, empathetic, and very empathetic) on a scale of 1–5. Notably, these quality and empathy rating assessors evaluate the quality and empathy of the AI results based solely on the AI output, without knowledge of the patient's complete clinical information, reference standard results, or the correctness of the AI output.

During the early clinical evaluation stage, patient satisfaction (not satisfied, slightly satisfied, moderately satisfied, satisfied, and very satisfied) will be measured on a scale of 1–5 after AI responses have been generated. When evaluating satisfaction of the AI output, the patients are only aware of the AI output and remain uninformed about the reference standard results or the correctness of the AI output. After the evaluation, they can be informed of the reference standard results and the correctness of the AI output.

### Blinding

The blinding methods vary depending on the roles of the researchers and the stage of the study in which the researchers and subjects are involved. AI developers and AI output correctness assessors have access to complete patient information, including medical history, reference standard results, and AI output for determining correctness.

In our study, two roles are subject to blinding. (1) Reference standard assessors: These assessors possess knowledge of the patient's clinical information but are unaware of the AI output or its correctness. This approach helps mitigate information bias and classification bias. (2) Clinical researchers: These researchers are responsible for inputting data into the multimodal AI program. During the *in silico* development stage, they have access to all patient clinical information and reference standard results. However, during the silent evaluation and early clinical evaluation stages, they only have access to the patient's clinical information without knowledge of the reference standard. This approach helps mitigate diagnostic suspicion bias.

### Implementation

Clinical researchers responsible for AI system input data play a crucial role in influencing the research outcomes. Our implementation strategies focus on training researchers to minimize human-induced confounding bias. In the *in silico* development stage, researchers are required to hold a PhD in Ophthalmology from Fudan University and have at least 3 years of clinical experience as attending physicians. Prior to implementing the study, they must have completed rotational training in various ophthalmic subspecialties to ensure proficiency in medical inquiry, diagnostic criteria, and medical record documentation for common ophthalmic diseases.

During the silent evaluation and early clinical evaluation stages, clinical researchers will be junior university students specializing in clinical medicine at Fudan University. They will receive training in ophthalmology-related terminology and patient communication, enabling them to explain medical terms to patients and provide accurate responses to the AI system's queries. Data collection across the three research sites will be conducted by the same group of researchers.

For anterior segment image captured in medical facility, the technicians should possess a minimum of two years of experience to ensure precise lesion capture with appropriate lighting conditions. The researchers responsible for smartphone-based images should be pretrained to capture clear anterior segment images with smartphones.

### Statistical analysis

To assess the AI system's diagnostic and triage performance, we will use the one-vs.-rest strategy and calculate the area under the receiver-operating-characteristic curve (AUC), sensitivity, specificity, accuracy, positive predictive values and negative predictive values for disease diagnosis and subspecialty triage using IBM SPSS Statistics 26 software. Specifically, sensitivity measures the AI system's ability to correctly identify positive cases, such as diagnosing a specific disease or assigning a patient to the correct subspecialty. It is calculated by determining the ratio of true positives (correctly identified positive cases) to the sum of true positives and false negatives (missed positive cases). Conversely, specificity evaluates the AI system's capacity to accurately identify negative cases. For instance, it assesses the system's ability to correctly exclude the presence of a particular disease or to avoid assigning a patient to an incorrect subspecialty. Specificity is determined by calculating the ratio of true negatives (correctly identified negative cases) to the sum of true negatives and false positives (incorrectly identified negative cases). Accuracy, which indicates the proportion of correct classifications made by the AI system, will be determined as follows: Accuracy = (True positive + True negative)/(True positive + True negative + False positive + False negative). This parameter offers an overarching evaluation of the system's ability to make precise diagnoses and triage assignments. In addition to sensitivity, specificity, and accuracy, we will also calculate AUC, positive predictive values and negative predictive values. These parameters collectively provide a comprehensive assessment of the AI system's diagnostic and triage capabilities, ensuring a thorough evaluation of its performance. Subgroup analyses will be performed to explore the influence of lesion location (anterior segment disorders vs. posterior segment disorders) on the performance of the multimodal AI system. A *post-hoc* power calculation will also be conducted to assess how lesion locations influence the AI system's performance.

### Sample size calculation

In the *in silico* development stage, the sample size is adjusted based on AI system performance. Initially, each subspecialty involves 20 medical dialogues, with 80% for training ChatGPT and 20% for validation. If ChatGPT cannot generate inquiries, diagnoses, and triage results in the validation dataset, we'll increase the dialogues. Next, 50 virtual cases for the top 1–5 diseases in each subspecialty are input into the diagnostic system. If the sensitivity and specificity of specific diseases do not meet the predefined thresholds (sensitivity ≥ 90%, specificity ≥ 95%), we will proceed to develop a disease-specific image-based diagnostic system. For each disease, we will compile a dataset consisting of 50 cases, including both anterior segment images and their corresponding diagnoses. This dataset will be randomly partitioned into training and testing sets, with the training set supporting system development and the testing set assessing diagnostic accuracy. Should the predetermined criteria (sensitivity ≥ 90%, specificity ≥ 95%) be satisfied, there will be no necessity for further expansion of the sample size.

For the silent evaluation stage, the primary objective is to achieve a sensitivity ≥ 85% and specificity ≥ 95% for diagnosing ophthalmic diseases. To meet this goal, we set sensitivity = 0.85, specificity = 0.95, statistical power (1-β) = 85, significance level (α) = 0.05, and the sample size for each disease is determined to be 22, following the methodology (Tests for One-Sample Sensitivity and Specificity) outlined in Pass15. Since this stage covers at least four different illnesses, we will include a minimum of 88 patients in the entire sample. To account for potential confounding factors, we have opted to include 100 patients in this phase.

For the early clinical evaluation stage, all subjects collected within predefined date ranges at three sites will be included. Data collection at the Shanghai and Nanjing sites spans from July 21 to August 20, 2023. At the Suqian site, data collection is scheduled from August 21 to August 31, 2023.

### Data management

The research consortium organizes a central data management board, and the contact details of its members can be obtained from the consortium coordinator, Prof. Chen Zhao. Electronic medical records data for all recruited subjects will be securely stored in password-protected files on the hospital's local server. Access to these files will be restricted to researchers with the explicit consent of the subjects. Survey data, both input and output from the multimodal AI system, will be stored in a secure public server (aliyun.com). To safeguard patient privacy, we will not collect any Protected Health Information (PHI), including the patient's name, date of birth, and medical insurance number. The anterior segment images captured by either slit-lamp or smartphones will undergo digital masking to irreversibly remove identifiable features, while preserving disease-relevant features essential for diagnosis. To further ensure data security, clinical researchers, trained in data management, will utilize password-protected smartphones for data entry. The smartphone is securely stored in a cabinet with password protection and is not permitted to be taken outside of the research site. Data integrity will be rigorously maintained through a variety of mechanisms, including referential data rules, valid values, range checks, and consistency checks against data already stored in the database.

### Adverse event monitoring

The multimodal AI system doesn't disrupt standard patient management procedures, ensuring the integrity of diagnosis and treatment. Patients in the study also benefit from thorough medical history reviews by five expert clinicians, enhancing the standardization of their care. The major adverse events may stem from biased outputs and misinformation produced by language models. All participants (including researchers) in this study are encouraged to report any toxic information generated by the multimodal AI system, such as unhelpful information that fails to assist users in their tasks, dishonest information that fabricates or misleads users, and harmful information that can lead to physical, psychological, or social harm to individuals or the environment. In our prompt engineering process, we will include a fine-tuning phase to iteratively enhance content quality. Collecting toxic content is helpful for identifying areas for improvement and making necessary adjustments. Therefore, the fine-tuning process based on toxic content may facilitate adapting our AI system to real-world use and mitigating potential harms.

## Discussion

Our study aims to develop a multimodal AI system based on ChatGPT-3.5 and explore its performance for the diagnosis and triage of ophthalmic diseases. Existing alternative proprietary LLMs include ChatGPT-4.0 and Google Bard (Lim et al., 2023). Additionally, there are some open source LLMs that can be locally deployed. As we plan to apply our designed multimodal AI system in clinical experiments, it necessitates swift interactive responses and the ability to handle parallel tasks. There are two primary reasons for not utilizing locally deployed LLM models. Firstly, their text generation performance is not as robust as ChatGPT, which hinders rapid interaction and concurrent conversations. Secondly, it demands a substantial amount of data for initial local training, which doesn't align with our objective of rapid product development and application in clinical experiments. ChatGPT currently stands out as the most proficient and reliable LLM product on the market for interactive applications. While ChatGPT-4.0 lacks support for prompt engineering, and Google Bard was not yet available at the outset of our research, we made the decision to develop our multimodal AI system based on ChatGPT-3.5.

One of the strengths of our multimodal AI system is its applicability to different ophthalmic diseases. Previous studies predominantly rely on photographs to detect certain ocular and visual abnormalities (Ting et al., 2019; Tan et al., 2021; Tiwari et al., 2022; Lou et al., 2023; Shao et al., 2023a). These image-centric technologies, while valuable, have limitations when it comes to large-scale applications across diverse ophthalmic conditions. The limitations primarily stem from the absence of comprehensive medical histories, which are crucial for accurate diagnosis (Ting et al., 2019; Moor et al., 2023). Therefore, we aim to integrate medical history inquiry with image-based diagnosis to empower a multimodal AI system. According to the protocol, we will evaluate the performance of this system in detecting most of the common ophthalmic diseases, including anterior and posterior segment disorders, strabismus, neoplasms, aesthetic abnormalities and ocular manifestations of systemic diseases. This system is efficient for triage procedures. During registration, pre-registration desk staff can assist patients with AI-powered diagnosis and triage, guiding them to subspecialty appointments.

Another advantage of this multimodal AI system is its capability for medical history inquiries based on the patient's chief complaints. This form of inquiry, which is more structured than directly inputting symptom descriptions into ChatGPT, allows for logical and in-depth questioning centered around the symptoms presented by the patient (Howard et al., 2023). This technical advancement not only contributes to improved diagnostic accuracy but also aids physicians in the comprehensive documentation of medical history (Au and Yang, 2023). In clinical practice, physicians often rely on their own experiences during medical inquiry, potentially leading to biases toward conditions they have recently encountered or commonly come across (Rau et al., 2023). This bias can increase the risk of misdiagnosis, particularly in communities with limited access to ophthalmic resources. By utilizing this AI-based medical history collection framework, physicians can mitigate the impact of individual biases (Huang et al., 2023). The structured and systematic approach ensures that critical questions are asked and relevant information is captured, providing a more objective and comprehensive view of the patient's medical history.

Moreover, our study represents a significant effort to explore the reliability and feasibility of smartphone-based multimodal AI system for diagnosing and triaging ophthalmic diseases in real-world scenarios. As outlined in our protocol, the multimodal AI system will initially employ slit lamp-based anterior segment images in the silent evaluation stage and subsequently upgrade to using smartphone-based anterior segment images in the early clinical evaluation stage. This upgraded system is user-friendly, as it doesn't require specialized medical equipment (Chen et al., 2023b). A smartphone-based system to detect ocular pathology has obvious real-world applications. For instance, within local communities, the early detection of ocular abnormalities by this smartphone-based AI program can facilitate timely referrals to ophthalmologists and enable prompt intervention. This capability may improve vision-related outcomes and even survival rates in cases such as retinoblastoma (Chen et al., 2023b). Additionally, some patients with relatively minor or non-urgent conditions may have the opportunity to bypass direct physician evaluation and instead be referred for routine outpatient follow-ups at local community hospitals. This approach can support the implementation of a tiered healthcare system and alleviate the burden on tertiary healthcare facilities (Chen et al., 2023b).

Additionally, we will explore the quality and empathy of the answers generated by the AI system. Previous research has indicated that ChatGPT produces high-quality responses with a strong sense of empathy (Ayers et al., 2023; Janamla et al., 2023). This suggests that our system could be of assistance to clinicians when communicating with patients. It could help by composing a message based on a patient's query, which can then be reviewed and edited by physicians or support staff before sending. We also place significant importance on documenting potential toxic information. Large language models may produce outputs that are untruthful, toxic, or not useful to the user (Gilbert et al., 2023). There is a growing field dedicated to establishing benchmarks for a concrete assessment of these risks, focusing particularly on issues related to toxicity, stereotypes, and social bias. Recording such adverse events will aid in future efforts to modify the behavior of language models, with the goal of mitigating these harms.

AI-assistant system has the potential to significantly impact patient outcomes (Liu et al., 2023). By providing quick, empathetic, and high-quality responses to patients' inquiries, it has the potential to reduce unnecessary clinical visits, thereby freeing up valuable healthcare resources for those who truly require them. Furthermore, the high-quality responses generated by AI system can positively influence patient health behaviors, including medication adherence, compliance with daily monitoring, and fewer missed appointments (Rompianesi et al., 2022). We are looking forward to conducting randomized clinical trials to assess the effects of implementing this multimodal AI-assistant system on patient outcomes, as well as its potential socioeconomic benefits.

## Limitation

This study protocol has several limitations. First, there is a potential for Berkson bias due to variations in patient sources across the three medical centers, resulting in variations in the basic characteristics of the subjects. This includes differences in the proportions of specialist ophthalmic diseases among the patient populations. Additionally, there is a risk of information bias. Patients intentionally exaggerating or downplaying their symptoms can introduce reporting bias, while inconsistencies in the measurement standards of different investigators may lead to investigation bias. Moreover, factors such as environmental interference and variations in imaging quality (including blurring, brightness, and pixel differences) may impact the system's performance and therefore limit the generalizability of this AI system. There may be certain restrictions on transferring this multimodal AI system to community hospitals and even at-home settings. It's important to note that our study might not detect rare diseases due to limited sample size. Further evidence is required to support the application of this AI system for diagnosing rare ophthalmic conditions.

Furthermore, it's important to consider that ChatGPT, being a proprietary (closed source) model, is susceptible to unannounced alterations, potentially impacting the diagnostic efficacy of our system. As per a report by L. Chen et al., ChatGPT-3.5 in its June 2023 release exhibited a decline in accuracy within the LangChain HotpotQA Agent domain and the Code Generation and Formatting domain when compared to ChatGPT-3.5 from March 2023 (Chen et al., 2023a). However, it's noteworthy that within the USMLE Medical Exam domain, ChatGPT-3.5 (June 2023) continued to demonstrate commendable accuracy. This suggests that a medical diagnostic system developed on the foundations of ChatGPT may maintain diagnostic performance within the medical field. Careful consideration of these observations is crucial as we advance our AI system for ophthalmic disease diagnosis and triage.

## Ethics statement

The studies involving humans were approved by Fudan Eye & ENT Hospital Institutional Review Board. The studies were conducted in accordance with the local legislation and institutional requirements. The participants provided their written informed consent to participate in this study.

## Author contributions

ZP: Methodology, Writing—original draft. RM: Methodology, Writing—original draft. YiZ: Writing—original draft. MY: Data curation, Writing—original draft. JLu: Data curation, Writing—original draft. QC: Investigation, Software, Writing—review & editing. JLi: Investigation, Software, Writing—review & editing. YuZ: Formal analysis, Writing—review & editing. JW: Data curation, Writing—review & editing. YZ: Writing—review & editing. JZ: Writing—review & editing. BQ: Writing—review & editing. QJ: Writing—review & editing. FS: Methodology, Software, Writing—review & editing. JQ: Investigation, Writing—review & editing. XC: Investigation, Software, Writing—review & editing. CZ: Writing—original draft, Writing—review & editing.
