# Supplementary material for: Development and evaluation of multimodal AI for diagnosis and triage of ophthalmic diseases using ChatGPT and anterior segment images: protocol for a two-stage cross-sectional study
Source: Front Artif Intell. 2023 Dec 8;6:1323924. doi: 10.3389/frai.2023.1323924 (PMC10748413; doi:10.3389/frai.2023.1323924)
Supplement: Supplementary file 1 [file Table_1.DOCX]

| **Table 1. Triage during the in silico development stage** | |
| --- | --- |
| general ophthalmology | glaucoma |
| refractive diseases | retina |
| strabismus | neuro-ophthalmology |
| cornea | oculoplastics and orbital diseases |
| cataract | ophthalmic emergencies |

Supplementary Table 1. Triages during the in silico development stage.

| **Table 2. Diagnosis during the in silico development stage** | | |
| --- | --- | --- |
| acute angle closure glaucoma | endophthalmitis | ocular contusion |
| allergic conjunctivitis | epicanthus | ocular hypertension |
| amblyopia | epimacular membrane | ophthalmic physical examination |
| ametropia | esotropia | optic atrophy |
| angle recession | exotropia | optic neuritis |
| asthenopia | eyelid inflammation | orbital contusion |
| astigmatism | eyelid tumor | orbital fracture |
| blepharitis | foreign body in conjunctival sac | orbital tumor |
| blepharochalasis | glaucoma | palpebral edema |
| blepharospasm | Graves orbitopathy | pigmented nervus of conjunctiva |
| cataract | hemicrania | post-cataract surgery |
| chalazion | hypermetropia | post-corneal surgery |
| conjunctival chemosis | hypochromatopsia | presbyopia |
| conjunctival lithiasis | intraocular hemarrhage | pterygium |
| conjunctival scar | intraocular tumor | ptosis |
| conjunctival tumors | iridocyclitis | retinal detachment |
| conjunctivitis | iritis | retinal disorders |
| corneal defect | ischemic optic neuropathy | retinal edema |
| corneal edema | keratitis | retinal vein obstruction |
| corneal epithelial defect | keratoconus | scleritis |
| corneal foreign body | keratohelcosis | strabismus |
| corneal scar | laceration of eyelid | subconjunctival hemorrhage |
| cyanosis syndrome | lacrimal duct obstruction | suture exposure |
| dacryocanaliculitis | lens dislocation | trichiasis |
| dacryocystitis | macular degeneration | uveitis |
| diabetic retinopathy | macular hole | vitreous opacity |
| dry eyes | macular oedema |  |
| endophthalmitis | myopia |  |

Supplementary Table 2. Clinical diagnosis during the in silico development stage.

| **Table 3. Diagnosis during the silent evaluation stage** |
| --- |
| eyelid tumor |
| trichiasis |
| ptosis |
| iridocyclitis |
| cyanosis syndrome |
| scleritis |
| angle-cosure glaucoma |
| pterygium |
| senile cataract |
| keratitis |
| keratohelcosis |

Supplementary Table 3. Clinical diagnosis during the silent evaluation stage.
