# Supplementary material for: Development and evaluation of multimodal AI for diagnosis and triage of ophthalmic diseases using ChatGPT and anterior segment images: protocol for a two-stage cross-sectional study
Source: Front Artif Intell. 2023 Dec 8;6:1323924. doi: 10.3389/frai.2023.1323924 (PMC10748413; doi:10.3389/frai.2023.1323924)
Supplement: Supplementary file 3 [file Table_3.DOC]

**Multimodal Machine Learning in Ophthalmic Diseases: Informed Consent**

Dear Sir/Madam,

We would like to invite you to participate in a clinical study entitled "Multimodal Machine Learning in Ophthalmic Diseases". Before you decide to participate in this study, please carefully read this informed consent form and make a well-considered decision. You may ask your research physician any questions you do not understand and continue your discussion until you are fully informed. If you are participating in other research, please inform your research physician. The main points of this study are as follows:

**1. Study Background**

Nature of the Project: The rapid development of multimodal machine learning technology has provided new possibilities for intelligent ophthalmology diagnosis. This study aims to collect subject medical history and eye image data intelligently through a mini-program, followed by clinical diagnosis and treatment provided by clinical doctors to achieve intelligent ophthalmic disease diagnosis. After multimodal machine learning training, intelligent diagnosis of ophthalmic diseases is enabled. This study plans to prospectively enroll 2000 subjects with eye complaints and 200 control subjects without eye complaints, using clinical diagnosis as the gold standard, to analyze the assessment performance and application value of multimodal machine learning for intelligent ophthalmic disease diagnosis.

Implementation: The study is led by the Eye, Ear, Nose and Throat Hospital affiliated with Fudan University and jointly conducted with the Eye Hospital affiliated with Nanjing Medical University and the First People's Hospital of Suqian. The project will commence on July 1, 2023. After subjects are enrolled, they will undergo intelligent inquiry and data collection via a mobile application, and subjects with eye complaints will be diagnosed by clinical doctors to obtain the final diagnosis.

Ethical Principles: This study will follow international principles that protect the rights of subjects, such as the Helsinki Declaration, as well as relevant national laws and regulations of China, in accordance with medical ethics.

**2. Study Design and Procedures**

Study Procedures: If you decide to participate in this study, you will link to the application through methods such as scanning a QR code or searching and proceed to fill out the questionnaire. After completion, the system will generate intelligent diagnostic results. These results will be kept confidential between you and the clinical doctor and can only be accessed by the research staff of this project.

Personal Privacy Protection Measures: The information you provide will only be available for the research staff to access. When necessary, government regulatory authorities or members of ethics committees can access your personal information as required by regulations. Research results will be published in the form of statistically analyzed data and will not contain any identifiable subject information.

**3. Potential Risks and Benefits**

Potential Risks: When using the mobile application, there may be risks of leakage or unauthorized access to the patient's personal medical information and image data. To address this issue, we will ensure subject information security through user authentication, data access control, data transmission encryption, security audits, and monitoring.

Potential Benefits: Participation in this study offers no economic or specific therapeutic benefits. We extend our gratitude to you for contributing to medical development.

**4. Voluntary Participation**

Your participation in the study is entirely voluntary. You may withdraw from the study at any time without providing a reason. It will not affect your relationship with healthcare providers and your future treatment.

**5. Study Costs**

Participation in this study will not incur any additional costs for you, nor will you receive any financial benefits.

**6. Contact Information**

If you have any questions about this study, you may directly contact Dr. Ma Ruiqi at the Eye, Ear, Nose and Throat Hospital affiliated with Fudan University. Contact Phone: 021-64377134.

**7. Signature of the Informed Consent Form**

I have read the above information, understand the purpose of this study, and the potential benefits of participating in this study. I have had all my questions about the study procedures and content satisfactorily answered. I voluntarily sign this informed consent form and agree to participate in this study.

Participant's Signature: [Your Signature]

Date of Signature:

We have read and explained this informed consent form to the study subject and have answered all questions raised by him/her. The subject has understood and agreed to participate in this scientific study.

Researcher's Signature: [Researcher's Signature]

Date of Signature:

**眼科疾病辅助诊断的多模态机器学习效能评估及临床应用**

**受试者知情同意书**

尊敬的先生/女士：

我们将邀请您参加一项临床研究，题目是“眼科疾病辅助诊断的多模态机器学习效能评估及临床应用”。在您参加此项研究之前，请您仔细阅读这份知情同意书并慎重做出是否参加本项研究的决定。您可以向您的研究医生询问任何您不懂的地方，让他给您解释，直到您完全理解为止。您在做出参加此项研究的决定之前，可以和您的家人及朋友进行充分的讨论。若您正在参加别的研究，请告知您的研究医生。本研究的主要内容如下：

一、研究背景

1. 课题的性质：多模态机器学习技术的快速发展，为解决眼科智能诊断提供了新的可能性。本研究拟通过小程序智能收集受试者的病史信息和眼部图像数据，进而由临床医生为受试者提供眼科临床诊疗并明确最终诊断。通过多模态机器学习训练后，实现眼科疾病的智能诊断。本课题拟前瞻性入组2000例有眼部主诉的受试者、200例无眼部主诉的对照者，以临床诊断为金标准，分析多模态机器学习对眼科疾病智能诊断的评估效能和应用价值。

2. 本研究由复旦大学附属眼耳鼻喉科医院牵头，联合南京医科大学附属眼科医院、宿迁市第一人民医院共同实施。课题起始时间为2023年7月1日，受试者入组后将在手机应用程序上进行智能问诊和图片资料收集，有眼部主诉的受试者将在临床医生处进行就诊并得到最终诊断。

3. 此项研究将遵从赫尔辛基宣言等保护受试者权益的国际原则和中国国家相关法律、法规，符合医学伦理道德。

二、研究设计和研究过程

1. 研究过程**：**如果您决定参加本项研究，您将通过扫码、搜索等方式链接到应用程序并进行填写。填写完成后，系统将生成智能诊断结果，该结果会对您和临床医生保密，仅可由本课题的研究人员查阅。

2. 个人隐私的保密措施：您填写的相关资料仅供研究人员查阅。必要时，政府管理部门或伦理委员会的成员按规定可以查阅您的个人资料。研究结果将以经统计分析后的数据形式发表，不包含任何可识别的受试者信息。

三、可能的风险和受益：

1. 可能的风险：在使用手机应用程序时，患者的个人医疗信息和图像数据可能会面临泄露或未经授权访问的风险。针对该问题，我们将采用用户身份验证、数据访问权限控制、数据传输加密、安全审计及监控等方式保证受试者的信息安全。

2．可能的受益：参与本项研究无经济受益，也无明确的疗效受益。在此我们为您对医学发展所做出的贡献表示感谢！

四、自愿参加：您参与研究是完全自愿的。您可以随时退出研究而无需理由。绝不会影响您和医务人员的关系及今后的诊治。

五、研究费用：参加此项研究不会增加您的任何费用，但也无任何经济受益。

六、联系人及联系方式：如果您本人对此项研究有任何疑问，可直接与复旦大学附属眼耳鼻喉科医院马睿琦医生联系。联系电话：021-64377134。

七、该知情同意书一式两份，双方妥善保管，在双方签字后有效。

**知情同意书签字处**

我已阅读上述信息并理解该研究的目的以及参加该研究所带来的潜在益处，我对研究程序、研究内容提出的所有问题均已得到令我满意的答复。我自愿签署这份知情同意书，并自愿参加这项研究。

参加者签字： 签字日期：

参加者联系电话：

我们已经向研究对象宣读和解释了这份知情同意书，并且回答了他/她所提出的所有问题。他/她本人也已经理解并同意参加此项科学研究。

研究者签字： 签字日期：
